# *In Vitro* Activity of Rifampin, Rifabutin, and Rifapentine against Enterococci and Streptococci from Periprosthetic Joint Infection

**DOI:** 10.1128/spectrum.00071-21

**Published:** 2021-07-14

**Authors:** Mariana Albano, Melissa J. Karau, Kerryl E. Greenwood-Quaintance, Douglas R. Osmon, Caitlin P. Oravec, Daniel J. Berry, Matthew P. Abdel, Robin Patel

**Affiliations:** a Division of Clinical Microbiology, Department of Laboratory Medicine and Pathology, Mayo Clinic, Rochester, Minnesota, USA; b Division of Infectious Diseases, Department of Medicine, Mayo Clinic, Rochester, Minnesota, USA; c Department of Orthopedic Surgery, Mayo Clinic, Rochester, Minnesota, USA; University of Arizona/Banner Health

**Keywords:** rifamycin, periprosthetic joint infection, streptococci, enterococci, biofilm, rifampin, rifabutin, rifapentine

## Abstract

After staphylococci, streptococci and enterococci are the most frequent causes of periprosthetic joint infection (PJI). MICs and minimum biofilm bactericidal concentrations of rifampin, rifabutin, and rifapentine were determined for 67 enterococcal and 59 streptococcal PJI isolates. Eighty-eight isolates had rifampin MICs of ≤1 μg/ml, among which rifabutin and rifapentine MICs were ≤ 8 and ≤4 μg/ml, respectively. There was low rifamycin *in vitro* antibiofilm activity except for a subset of Streptococcus mitis group isolates.

**IMPORTANCE** Rifampin is an antibiotic with antistaphylococcal biofilm activity used in the management of staphylococcal periprosthetic joint infection with irrigation and debridement with component retention; some patients are unable to receive rifampin due to drug interactions or intolerance. We recently showed rifabutin and rifapentine to have *in vitro* activity against planktonic and biofilm states of rifampin-susceptible periprosthetic joint infection-associated staphylococci. After staphylococci, streptococci and enterococci combined are the most common causes of periprosthetic joint infection. Here, we investigated the *in vitro* antibiofilm activity of rifampin, rifabutin, and rifapentine against 126 Streptococcus and *Enterococcus* periprosthetic joint infection isolates. In contrast to our prior findings with staphylococcal biofilms, there was low antibiofilm activity of rifampin, rifabutin, and rifapentine against PJI-associated streptococci and enterococci, apart from some Streptococcus mitis group isolates.

## OBSERVATION

Rifampin is an antibiotic with antibiofilm activity used in the management of staphylococcal periprosthetic joint infection (PJI) with irrigation and debridement with component retention (IDCR) (1, 2); some patients are unable to receive rifampin due to drug interactions or intolerance. We recently showed that rifabutin and rifapentine, which have more favorable drug interaction/side effect profiles than rifampin, have *in vitro* activity against planktonic and biofilm states of rifampin-susceptible PJI-associated staphylococci (3), and that these rifamycins are as active as rifampin in combination therapy regimens in experimental rat Staphylococcus aureus foreign body osteomyelitis (4). After staphylococci, streptococci and enterococci combined are the most common causes of PJI, accounting for up to 20% of cases (5–8). Here, we investigated the *in vitro* activity of rifampin, rifabutin, and rifapentine, alongside levofloxacin, against planktonic and biofilm states of Streptococcus and *Enterococcus* PJI isolates.

The *in vitro* activity of rifampin, rifabutin, rifapentine, and levofloxacin against planktonic and biofilm states of 126 Streptococcus and *Enterococcus* PJI isolates was tested. Isolates were collected between 1996 and 2018 from separate patients with infected arthroplasties managed at the Mayo Clinic and included 61 isolates of E. faecalis, 6 E. faecium, 23 S. agalactiae, 1 S. pyogenes, 6 S. dysgalactiae, 17 S. mitis group, 6 *S. anginosus* group, 4 *S. salivarius* group, 1 S. mutans group, and 1 S. gallolyticus. E. faecalis ATCC 29212 and S. pneumoniae ATCC 49619 were used as quality control strains. Rifampin, rifabutin, rifapentine, and levofloxacin (Sigma-Aldrich, St. Louis, MO) MICs were determined by broth microdilution by following Clinical and Laboratory Standards Institute (CLSI) guidelines (9, 10). Rifampin and levofloxacin were prepared following CLSI guidelines (10). Rifabutin and rifapentine were prepared in dimethyl sulfoxide and methanol, respectively, per the manufacturer’s instructions. Current CLSI rifampin breakpoints for enterococci are ≤1  μg/ml susceptible, 2  μg/ml intermediate, and ≥4  μg/ml resistant. There are no rifampin breakpoints defined by the CLSI for beta-hemolytic or viridans group streptococci. No rifabutin or rifapentine breakpoints are defined for any of the tested bacteria (10). EUCAST rifampin breakpoints for Streptococcus groups A, B, C, and G are ≤0.06  μg/ml susceptible and >0.5  μg/ml resistant, and the EUCAST epidemiological cutoff (ECOFF) for viridans group streptococci is 0.125  μg/ml (11). Levofloxacin breakpoints defined by CLSI for all organism types tested are ≤2  μg/ml susceptible, 4  μg/ml intermediate, and ≥8  μg/ml resistant (10). Minimum biofilm bactericidal concentration (MBBC) values were determined using a pegged-lid microtiter plate assay, as previously described (3, 12).

Detailed findings for all study isolates are depicted in Table S1 in the supplemental material, which shows the aggregated MIC and MBBC values for the E. faecalis, S. agalactiae, and S. mitis group isolates. Overall, 29/61 (48%) E. faecalis isolates were rifampin susceptible, among which rifabutin and rifapentine MICs were ≤8 and ≤4  μg/ml, respectively (Table 1). All enterococcal rifamycin MBBCs were >8  μg/ml, except for E. faecalis IDRL-11962 (all rifamycin MBBCs, 4  μg/ml). Overall, 48/61 (79%) E. faecalis isolates were levofloxacin susceptible; levofloxacin MBBCs were >8  μg/ml, except for E. faecalis IDRL-10026 (levofloxacin MBBC, 4  μg/ml) (Table 1).

**TABLE 1:** E. faecalis (*n*  =  61), S. agalactiae (*n*  =  23), and S. mitis group (*n*  =  17) rifampin, rifabutin, rifapentine, and levofloxacin MICs and MBBCs

Parameter	Drug	No. of isolates (cumulative %) with the following value (μg/ml):									MIC_50_ (μg/ml)	MIC_90_ (μg/ml)	MBBC_50_ (μg/ml)	MBBC_90_ (μg/ml)
		0.03	0.06	0.125	0.25	0.5	1	2	4	≥8				
E. faecalis														
MIC	Rifampin			2 (3)	3 (8)	13 (30)	11 (48)	14 (70)	11 (89)	7 (100)	2	≥8		
	Rifabutin			1 (2)	3 (7)	4 (13)	11 (31)	6 (41)	15 (66)	21 (100)	4	≥8		
	Rifapentine				1 (2)	4 (8)	6 (18)	18 (48)	20 (80)	12 (100)	4	≥8		
	Levofloxacin				3 (5)	7 (16)	27 (61)	11 (79)	1 (80)	12 (100)	1	≥8		
MBBC	Rifampin								1 (2)	60 (100)			≥8	≥8
	Rifabutin								1 (2)	60 (100)			≥8	≥8
	Rifapentine								1 (2)	60 (100)			≥8	≥8
	Levofloxacin								1 (2)	60 (100)			≥8	≥8
S. agalactiae														
MIC	Rifampin		3 (13)	5 (35)	15 (100)						0.25	0.25		
	Rifabutin		7 (30)	10 (74)	6 (100)						0.125	0.25		
	Rifapentine			3 (13)	5 (35)	14 (96)	1 (100)				0.5	0.5		
	Levofloxacin					10 (43)	11 (91)	2 (100)			1	1		
MBBC	Rifampin									23 (100)			≥8	≥8
	Rifabutin									23 (100)			≥8	≥8
	Rifapentine									23 (100)			≥8	≥8
	Levofloxacin						1 (4)		4 (22)	18 (100)			≥8	≥8
S. mitis group														
MIC	Rifampin	5 (31)	8 (81)	1 (87)	1 (94)				1 (100)		0.06	0.125		
	Rifabutin	3 (19)	6 (56)	6 (94)						1 (100)	0.06	0.125		
	Rifapentine		4 (25)	6 (62)	4 (87)	1 (94)			1 (100)		0.125	0.25		
	Levofloxacin					6 (37)	9 (94)	1 (100)			1	1		
MBBC	Rifampin		2 (12)	2 (24)	1 (29)				1 (35)	11 (100)			≥8	≥8
	Rifabutin	1 (6)	3 (24)			1 (29)	5 (59)		3 (76)	4 (100)			1	≥8
	Rifapentine			2 (12)	3 (29)	1 (35)	1 (41)			10 (100)			≥8	≥8
	Levofloxacin					1 (6)	8 (53)	3 (71)	3 (88)	2 (100)			1	4

All 23 S. agalactiae isolates tested had rifampin MICs of ≤0.25  μg/ml (among which 3 would be considered susceptible and 20 intermediate by EUCAST breakpoints), with rifabutin and rifapentine MICs of ≤0.25 and ≤1  μg/ml, respectively (Table 1). All 6 S. dysgalactiae isolates had rifampin MICs of 0.03  μg/ml (susceptible based on EUCAST breakpoints), among which rifabutin and rifapentine MICs were 0.03 and ≤0.06  μg/ml, respectively (Table S1). S. agalactiae and S. dysgalactiae rifamycin MBBCs were all >8  μg/ml (Table S1).

S. mitis group isolates had rifampin, rifabutin, and rifapentine MICs of ≤0.25, ≤0.125, and ≤0.5  μg/ml, respectively, except one isolate, which had MICs of 4, >8, and 4  μg/ml, respectively; 87% of these isolates were at or below the EUCAST rifampin ECOFF (Table 1). MBBC_50_ values for rifampin, rifabutin, and rifapentine were ≥8, 1, and ≥8  μg/ml, respectively.

All six *S. anginosus* group isolates tested had rifampin MICs of ≤0.5  μg/ml, rifabutin MICs of ≤0.5  μg/ml, and rifapentine MICs of ≤1 μg/ml (Table S1). Four isolates were at or below the EUCAST rifampin ECOFF. All *S. anginosus* group rifamycin MBBCs were >8  μg/ml, except for IDRL-12364 (rifabutin MBBC, 0.5  μg/ml).

All but one of the streptococcal isolates tested were levofloxacin susceptible. For S. agalactiae, levofloxacin MBBCs were >8  μg/ml for 18/23 isolates (Table 1). For levofloxacin-susceptible S. dysgalactiae, levofloxacin MBBCs were 4  μg/ml for 2 isolates and >8  μg/ml for 4 isolates (Table S1). For *S. anginosus* group, levofloxacin MBBCs were 2  μg/ml for 2 isolates and ≥8  μg/ml for 4 isolates (Table S1). For S. mitis group isolates, levofloxacin MBBCs were ≥1  μg/ml (Table 1).

In contrast to our findings with staphylococcal biofilms (3), results of this study show low *in vitro* activity of rifamycins against enterococcal biofilms. The biofilm results reported here are consistent with those of other reports. Holmberg et al. studied rifampin and ciprofloxacin alone and in combination against 15 PJI E. faecalis isolates (13). All except one isolate was rifampin susceptible, but MBBCs (tested for four isolates) were 64 to 128  μg/ml (13). Likewise, for ciprofloxacin, three isolates had ciprofloxacin MICs of >16  μg/ml, with the remaining classified as susceptible; ciprofloxacin MBBCs (tested for four isolates) were 256  μg/ml (13). This is similar to our findings with levofloxacin. Holmberg et al. also reported rifampin MICs of 1 to 2  μg/ml and MBBCs of 64 to 128  μg/ml for three E. faecium PJI isolates (14). Minardi et al. reported planktonic MICs of 2  μg/ml for E. faecalis ATCC 29212 and ATCC 51299, with adherent biofilm concentrations of 16 and 32  μg/ml, respectively, using a crystal violet assay (15). They evaluated tigecycline and rifampin alone or combined for prevention of ureteral stent infection in an experimental rat model, showing more activity of combination therapy than either drug alone (13, 15). Oliva et al. showed that rifampin alone had no activity against enterococcal biofilms, either *in vitro* or *in vivo*, but did demonstrate activity when administered as a combination therapy (16).

Data on rifampin treatment of enterococcal PJI is sparse. Thompson et al. reported a tendency toward better outcome with rifampin-combination therapy for enterococcal PJI; however, most cases were given combination therapy directed toward coinfections with staphylococci (17). Tornero et al. reviewed characteristics and outcomes of 203 patients with enterococcal PJI at 18 hospitals in 6 European countries. For those with infection within 30  days of implantation, rifampin in combination with another active antibiotic was associated with a higher remission rate than alternatives without rifampin (18).

Fiaux et al. reported results of a retrospective multicenter cohort study of 95 streptococcal PJIs from 2001 through 2009 (19). All isolates tested were rifampin susceptible. Fifty-five cases were treated with IDCR with rifampin combinations, including with levofloxacin, used in 52 and 28 cases, respectively; the overall remission rate was 71%. Antibiotic treatment regimens other than rifampin combinations were associated with worse outcome by univariate analysis (19). Rifampin combinations, including with levofloxacin, were associated with improved remission rates. Andronic et al. found no difference in failure rates with or without rifampin in a retrospective analysis of 22 streptococcal PJIs from a single institution, five of which were treated with rifampin combination regimens (20). In a study by Loubet et al. that included six S. agalactiae PJI cases, two were treated with combinations with rifampin, one with a good outcome; however, only 57% of tested S. agalactiae strains were susceptible to rifampin (21). Lora-Tamayo et al. recently published results of a retrospective, observational, multicenter, international study of 462 streptococcal PJI cases managed with IDCR, 37% of which were managed with rifampin. Failure occurred in 42% (187/444) of evaluable patients. Early use of rifampin and treatment for ≥21  days with a β-lactam as monotherapy or in combination with rifampin was associated with successful outcomes (22). The relevance of *in vitro* biofilm susceptibility testing and its relationship with clinical success with combination rifamycin therapies is incompletely defined.

This study is one of the largest evaluating the *in vitro* planktonic and biofilm activity of rifampin against PJI-associated streptococci and enterococci and, to our knowledge, the only study evaluating rifabutin and rifapentine against PJI isolates. Overall, there was low antibiofilm activity of rifamycins against PJI-associated streptococci and enterococci, with the exception of some S. mitis group isolates. Whether the study findings correlate with *in vivo* efficacy or *in vitro* efficacy in combination with other agents remains to be determined.
