# Supplementary material for: In Vitro Activity of Rifampin, Rifabutin, and Rifapentine against Enterococci and Streptococci from Periprosthetic Joint Infection
Source: Microbiol Spectr. 2021 Jul 14;9(1):10.1128/spectrum.00071-21. doi: 10.1128/spectrum.00071-21 (PMC8552655; doi:10.1128/spectrum.00071-21)
Supplement: SUPPLEMENTAL FILE 1 — Supplemental material. Download SPECTRUM00071-21_Supp_1_seq3.docx, DOCX file, 0.04 MB [file spectrum00071-21_supp_1_seq3.docx]

| Supplemental Table. Minimum inhibitory concentration (MIC) and minimum biofilm bactericidal concentration (MBBC) of rifampin, rifabutin, rifapentine and levofloxacin for 125 *Enterococcus* and *Streptococcus* species isolates. | | | | | | | | | |
| --- | --- | --- | --- | --- | --- | --- | --- | --- | --- |
| **Species** | **IDRL Isolate Number** | **Rifampin** | | **Rifabutin** | | **Rifapentine** | | **Levofloxacin** | |
|  |  | **MIC** | **MBBC** | **MIC** | **MBBC** | **MIC** | **MBBC** | **MIC** | **MBBC** |
| *Enterococcus faecalis* | 5985 | 0.5 | >8 | 0.5 | >8 | 0.5 | >8 | 4 | >8 |
|  | 6001 | 4 | >8 | ≥8 | >8 | 4 | >8 | 2 | >8 |
|  | 6028 | 8 | >8 | 4 | >8 | 8 | >8 | 0.5 | >8 |
|  | 6062 | 8 | >8 | >8 | >8 | 8 | >8 | 1 | >8 |
|  | 6080 | 8 | >8 | >8 | >8 | >8 | >8 | 2 | >8 |
|  | 6138 | 1 | >8 | 2 | >8 | 2 | >8 | >8 | >8 |
|  | 6171 | 1 | >8 | 4 | >8 | 2 | >8 | 1 | >8 |
|  | 6197 | 1 | >8 | 2 | >8 | 2 | >8 | 0.5 | >8 |
|  | 6198 | 1 | >8 | 4 | >8 | 4 | >8 | 1 | >8 |
|  | 6232 | 0.5 | >8 | 2 | >8 | 2 | >8 | 1 | >8 |
|  | 6233 | 0.5 | >8 | 2 | >8 | 2 | >8 | 1 | >8 |
|  | 6280 | 0.5 | >8 | 1 | >8 | 2 | >8 | >8 | >8 |
|  | 6301 | 0.5 | >8 | 1 | >8 | 2 | >8 | >8 | >8 |
|  | 7107 | 2 | >8 | 4 | >8 | 4 | >8 | 1 | >8 |
|  | 7120 | 2 | >8 | 4 | >8 | 4 | >8 | 2 | >8 |
|  | 7162 | 8 | >8 | 4 | >8 | 4 | >8 | 1 | >8 |
|  | 7241 | 1 | >8 | 1 | >8 | 2 | >8 | 2 | >8 |
|  | 7369 | 1 | >8 | 4 | >8 | 4 | >8 | 1 | >8 |
|  | 7370 | 2 | >8 | 4 | >8 | 4 | >8 | 1 | >8 |
|  | 7415 | 4 | >8 | 8 | >8 | 4 | >8 | 2 | >8 |
|  | 7548 | 2 | >8 | 2 | >8 | 4 | >8 | 1 | >8 |
|  | 7559 | 2 | >8 | 4 | >8 | 2 | >8 | 1 | >8 |
|  | 7582 | 4 | >8 | 8 | >8 | 8 | >8 | 1 | >8 |
|  | 7639 | 4 | >8 | >8 | >8 | 8 | >8 | 2 | >8 |
|  | 7722 | 0.125 | >8 | 0.25 | >8 | 0.5 | >8 | 0.5 | >8 |
|  | 8303 | 8 | >8 | 8 | >8 | 8 | >8 | >8 | >8 |
|  | 8451 | 4 | >8 | 8 | >8 | 4 | >8 | 1 | >8 |
|  | 8452 | 8 | >8 | >8 | >8 | >8 | >8 | 1 | >8 |
|  | 8455 | 4 | >8 | >8 | >8 | >8 | >8 | >8 | >8 |
|  | 8619 | 1 | >8 | 4 | >8 | 2 | >8 | 1 | >8 |
|  | 8620 | 2 | >8 | 8 | >8 | 8 | >8 | 1 | >8 |
|  | 9044 | 1 | >8 | 8 | >8 | 2 | >8 | 1 | >8 |
|  | 9065 | 0.5 | >8 | 1 | >8 | 1 | >8 | >8 | >8 |
|  | 9294 | 2 | >8 | 8 | >8 | 4 | >8 | 1 | >8 |
|  | 9634 | 2 | >8 | 8 | >8 | 4 | >8 | 1 | >8 |
|  | 9701 | 4 | >8 | >8 | >8 | 4 | >8 | 0.5 | >8 |
|  | 9761 | 0.25 | >8 | 0.125 | >8 | 2 | >8 | >8 | >8 |
|  | 10026 | 0.5 | >8 | 0.5 | 8 | 1 | >8 | 1 | 4 |
|  | 10256 | 2 | >8 | 8 | >8 | 4 | >8 | 1 | >8 |
|  | 10289 | 0.5 | >8 | 1 | >8 | 1 | >8 | >8 | >8 |
|  | 10948 | 4 | >8 | ≥8 | >8 | 8 | >8 | 1 | >8 |
|  | 11386 | 4 | >8 | 8 | >8 | ≥8 | >8 | 1 | >8 |
|  | 11569 | 4 | >8 | 8 | >8 | 4 | >8 | 1 | >8 |
|  | 11583 | 4 | >8 | 4 | >8 | 4 | >8 | 2 | >8 |
|  | 11592 | 0.125 | >8 | 0.5 | >8 | 0.5 | >8 | >8 | >8 |
|  | 11788 | 2 | >8 | 4 | >8 | 2 | >8 | 0.5 | >8 |
|  | 11857 | 1 | >8 | 0.25 | >8 | 1 | >8 | 0.5 | >8 |
|  | 11904 | 1 | >8 | 2 | >8 | 4 | >8 | 0.5 | >8 |
|  | 11911 | 0.5 | >8 | 1 | >8 | 1 | >8 | 1 | >8 |
|  | 11915 | 2 | >8 | 8 | >8 | 2 | >8 | 2 | >8 |
|  | 11962 | 0.25 | 4 | 0.25 | 4 | 0.25 | 4 | >8 | >8 |
|  | 11966 | 8 | >8 | 8 | >8 | 8 | >8 | 1 | >8 |
|  | 11967 | 2 | >8 | 4 | >8 | 4 | >8 | >8 | >8 |
|  | 11993 | 0.25 | >8 | 0.5 | >8 | 0.5 | >8 | 2 | >8 |
|  | 12011 | 1 | >8 | 1 | >8 | 2 | >8 | 1 | >8 |
|  | 12359 | 0.5 | >8 | 1 | >8 | 2 | >8 | 0.25 | >8 |
|  | 12361 | 0.5 | >8 | 1 | >8 | 2 | >8 | 0.25 | >8 |
|  | 12362 | 0.5 | >8 | 1 | >8 | 2 | >8 | 0.25 | >8 |
|  | 12374 | 0.5 | >8 | 1 | >8 | 1 | >8 | >8 | >8 |
|  | 12055 | 2 | >8 | 4 | >8 | 4 | >8 | 2 | >8 |
|  | 12062 | 2 | >8 | 4 | >8 | 4 | >8 | 2 | >8 |
| *Enterococcus faecium* | 8099 | 4 | >8 | 4 | >8 | 4 | >8 | 2 | >8 |
|  | 8305 | 0.03 | >8 | 0.03 | >8 | 0.03 | >8 | >8 | >8 |
|  | 8306 | 0.03 | >8 | 0.03 | >8 | 0.03 | >8 | >8 | >8 |
|  | 9066 | 4 | >8 | >8 | >8 | 8 | >8 | >8 | >8 |
|  | 11908 | >8 | >8 | >8 | >8 | >8 | >8 | >8 | >8 |
|  | 12056 | 4 | >8 | 4 | >8 | 4 | >8 | >8 | >8 |
| *Streptococcus agalactiae* | 5967 | 0.25 | >8 | 0.125 | >8 | 0.5 | >8 | 0.5 | 4 |
|  | 5968 | 0.25 | >8 | 0.125 | >8 | 0.5 | >8 | 0.5 | 4 |
|  | 5984 | 0.06 | >8 | 0.06 | >8 | 0.125 | >8 | 1 | >8 |
|  | 6087 | 0.25 | >8 | 0.25 | >8 | 0.5 | >8 | 0.5 | >8 |
|  | 6089 | 0.25 | >8 | 0.25 | >8 | 0.5 | >8 | 2 | >8 |
|  | 6144 | 0.125 | >8 | 0.125 | >8 | 0.5 | >8 | 0.5 | >8 |
|  | 6980 | 0.25 | >8 | 0.25 | >8 | 0.5 | >8 | 1 | >8 |
|  | 7200 | 0.06 | 8 | 0.06 | >8 | 0.125 | >8 | 0.5 | 1 |
|  | 7463 | 0.25 | >8 | 0.125 | >8 | 0.5 | >8 | 1 | >8 |
|  | 7515 | 0.25 | >8 | 0.125 | >8 | 0.5 | >8 | 1 | >8 |
|  | 7580 | 0.25 | >8 | 0.125 | >8 | 0.5 | >8 | 1 | >8 |
|  | 7656 | 0.25 | >8 | 0.25 | >8 | 0.5 | >8 | 1 | >8 |
|  | 8473 | 0.25 | >8 | 0.06 | >8 | 0.5 | >8 | 0.5 | 8 |
|  | 8557 | 0.125 | >8 | 0.06 | >8 | 0.25 | >8 | 0.5 | >8 |
|  | 9295 | 0.06 | >8 | 0.06 | >8 | 0.125 | >8 | 1 | >8 |
|  | 9433 | 0.25 | >8 | 0.125 | >8 | 0.5 | >8 | 1 | >8 |
|  | 9489 | 0.125 | >8 | 0.06 | >8 | 0.25 | >8 | 0.5 | 4 |
|  | 10197 | 0.25 | >8 | 0.25 | >8 | 0.5 | >8 | 0.5 | 4 |
|  | 10206 | 0.25 | >8 | 0.125 | >8 | 1 | >8 | 0.5 | >8 |
|  | 11503 | 0.25 | >8 | 0.125 | >8 | 0.25 | >8 | 1 | >8 |
|  | 11787 | 0.25 | >8 | 0.125 | >8 | 0.25 | >8 | 2 | >8 |
|  | 12018 | 0.125 | >8 | 0.06 | >8 | 0.25 | >8 | 1 | >8 |
|  | 12422 | 0.125 | >8 | 0.25 | >8 | 0.5 | >8 | 1 | >8 |
| *Streptococcus pyogenes* | 7467 | 0.03 | 8 | 0.03 | >8 | 0.06 | >8 | 0.5 | >8 |
| *Streptococcus dysgalactiae*  subspecies *canis* | 10052 | 0.03 | >8 | 0.03 | >8 | 0.06 | >8 | 0.5 | >8 |
|  | 11596 | 0.03 | >8 | 0.03 | >8 | 0.06 | >8 | 0.5 | >8 |
|  | 11801 | 0.03 | >8 | 0.03 | >8 | 0.06 | >8 | >8 | >8 |
|  | 11818 | 0.03 | >8 | 0.03 | >8 | 0.03 | >8 | 0.5 | >8 |
| *Streptococcus dysgalactiae* subspecies *equisimilis* | 7446 | 0.03 | >8 | 0.03 | >8 | 0.03 | >8 | 0.5 | 4 |
|  | 9069 | 0.03 | >8 | 0.03 | >8 | 0.03 | >8 | 0.5 | 4 |
| *S. mitis* group | 7601 | 0.06 | ≥8 | 0.125 | 1 | 0.125 | 8 | 2 | 2 |
|  | 8042 | 0.03 | 8 | 0.06 | 4 | 0.06 | 0.125 | 1 | 4 |
|  | 8782 | 0.06 | ≥8 | 0.125 | ≥8 | 0.25 | >8 | 0.5 | 1 |
|  | 10213 | 0.06 | ≥8 | 0.125 | 4 | 0.125 | >8 | 1 | 2 |
|  | 11469 | 0.06 | ≥8 | 0.125 | ≥8 | 0.125 | >8 | 1 | 8 |
|  | 11388 | 0.06 | ≥8 | 0.125 | 1 | 0.25 | >8 | 0.5 | 1 |
|  | 11492 | 0.06 | ≥8 | 0.06 | 1 | 0.125 | >8 | 0.5 | 1 |
|  | 11602 | 0.03 | 4 | 0.06 | 1 | 0.125 | 0.5 | 1 | 1 |
|  | 11570 | 0.25 | >8 | 0.06 | 0.5 | 0.5 | >8 | 1 | 2 |
|  | 12208 | 0.03 | 8 | 0.03 | 4 | 0.06 | >8 | 1 | 1 |
|  | 12274 | 0.06 | >8 | 0.03 | 0.06 | 0.25 | 0.25 | 1 | 4 |
|  | 5973 | 0.125 | 0.25 | 0.125 | 1 | 0.25 | 1 | 0.5 | 1 |
|  | 6122 | 4 | >8 | >8 | >8 | 4 | >8 | 0.5 | 8 |
|  | 7772 | 0.03 | 8 | 0.06 | >8 | 0.06 | >8 | 1 | 4 |
|  | 7423 | 0.06 | 0.125 | 0.06 | 0.06 | 0.06 | 0.25 | 1 | 1 |
|  | 6019 | 0.03 | 0.06 | 0.03 | 0.03 | 0.125 | 0.125 | 0.5 | 0.5 |
| *Streptococcus gallolyticus* | 6157 | 0.125 | >8 | 0.06 | 4 | 0.5 | >8 | 0.5 | >8 |
| *S. mutans* group | 7131 | 0.06 | 8 | 0.06 | >8 | 0.25 | >8 | 0.5 | >8 |
| *S. anginosus* group | 6079 | 0.06 | >8 | 0.06 | >8 | 0.25 | >8 | 1 | >8 |
|  | 11809 | 0.06 | >8 | 0.25 | >8 | 0.125 | >8 | 2 | >8 |
|  | 12266 | 0.25 | >8 | 0.25 | >8 | 0.5 | >8 | 0.5 | >8 |
|  | 6196 | 0.5 | >8 | 0.5 | >8 | 1 | >8 | 1 | 2 |
|  | 9298 | 0.125 | >8 | 0.03 | >8 | 0.25 | >8 | 0.5 | 8 |
|  | 12364 | 0.06 | >8 | 0.5 | 0.5 | 0.5 | >8 | 1 | 2 |
| *S. salivarius* group | 7527 | 0.06 | ≥8 | 0.06 | 8 | 0.125 | 4 | 2 | >8 |
|  | 11619 | 0.06 | ≥8 | 0.03 | 8 | 0.125 | 4 | 1 | 1 |
|  | 6131 | 0.125 | >8 | 0.06 | >8 | 0.125 | 4 | 1 | 2 |
|  | 6359 | 0.06 | 8 | 0.06 | 8 | 0.5 | >8 | 1 | 4 |
